# Prevalence of Heart Failure and Atrial Fibrillation in Minority Ethnic Subjects: The Ethnic-Echocardiographic Heart of England Screening Study (E-ECHOES)

**DOI:** 10.1371/journal.pone.0026710

**Published:** 2011-11-16

**Authors:** Paramjit S. Gill, Melanie Calvert, Russell Davis, Michael K. Davies, Nick Freemantle, Gregory Y. H. Lip

**Affiliations:** 1 Primary Care Clinical Sciences, University of Birmingham, Birmingham, United Kingdom; 2 University of Birmingham Centre for Cardiovascular Sciences City Hospital, Birmingham, United Kingdom; 3 University Hospitals Birmingham NHS Foundation Trust, Birmingham, United Kingdom; 4 Primary Care and Population Health, University College London, London, United Kingdom; Innsbruck Medical University, Austria

## Abstract

**Background:**

Limited data exists on the prevalence of heart failure amongst minority groups in the UK. To document the community prevalence and severity of left ventricular systolic dysfunction, heart failure, and atrial fibrillation, amongst the South Asian and Black African -Caribbean groups in the UK.

**Methods and Results:**

We conducted a cross-sectional study recruiting from September 2006 to July 2009 from 20 primary care centres in Birmingham, UK. 10,902 eligible subjects invited, 5,408 participated (49.6%) and 5,354 had complete data (49.1%). Subjects had median age 58.2 years (interquartile range 51.0 to 70.0), and 2544 (47.5%) were male. Of these, 1933 (36.3%) had BMI>30 kg/m^2^, 1,563 (29.2%) had diabetes, 2676 (50.0%) had hypertension, 307 (5.7%) had a history of myocardial infarction, and 104 (1.9%) had history of arrhythmia. Overall, 59 (1.1%) had an Ejection Fraction<40%, and of these 40 (0.75%) were NYHA class ≥2; 51 subjects (0.95%) had atrial fibrillation. Of the remaining 19 patients with an EF<40%, only 4 patients were treated with furosemide. A further 54 subjects had heart failure with preserved ejection fraction.

**Conclusions:**

This is the largest study of the prevalence of left ventricular systolic dysfunction, heart failure and atrial fibrillation in under-researched minority communities in the UK. The prevalence of heart failure in these minority communities appears comparable to that of the general population but less than anticipated given the high rates of cardiovascular disease in these groups. Heart failure continues to be a major cause of morbidity in all ethnic groups and preventive strategies need to be identified and implemented.

## Introduction

Heart failure (HF) is a major public health problem with global implications. The epidemiology of heart failure has been well characterised in the USA [1], [2], [3], [4] and Europe [5], [6] predominantly amongst the white population. Surveys in the United Kingdom (UK) and elsewhere report that 1–2% of the general population and 10–20% of the very elderly have HF [7], [8], [9]. However, limited data on ethnicity and heart failure are available outside North America and mainly amongst Black Americans. [10] Such information would inform healthcare provision as well as clinical management strategies, given the increasing number of ethnic minority groups in the UK. Further there is a need to increase data from minority groups in order to reduce racial and ethnic disparities in cardiovascular outcomes [11].

Heart failure directly accounts for 1.9% of total National Health Service (NHS) spending in the UK, with 69% of this being on hospitalisations, and indirectly (via long-term nursing care costs and secondary admissions) for a further equivalent of 2.0% of NHS expenditure [12]. Whilst there are well-established drug treatments for heart failure [1], [13], ethnic groups may respond differently to these therapies. [14], [15], [16] Further a large primary care based study in the UK, the Echocardiographic Heart of England Screening (ECHOES) study, reported that the prevalence of symptomatic left ventricular systolic dysfunction (LVSD) in a predominantly White population aged 45 and older was 0.96% [7].

There were 4.6 million people (7.9%) from the Black and minority ethnic groups in the 2001 UK Census, and the Black African-Caribbean, Indian, Pakistani and Bangladeshi groups comprised 2%, 1.8%, 1.3%, 0.5% respectively [17]. Importantly, cardiovascular morbidity and mortality are substantially higher amongst these ethnic groups than the White population. [17], [18] The prevalence of HF amongst these UK minority ethnic groups is currently not known as these groups have been underrepresented in previous studies [10].

The objective of the Ethnic-Echocardiographic Heart of England Screening study (E-ECHOES) was to establish the community prevalence and severity of LVSD and HF amongst the South Asian (SA) and Black African-Caribbean (AC) ethnic groups in the UK. Further objectives were to assess the prevalence of atrial fibrillation, and the differences, if any, in heart failure risk factors between SA and AC ethnic populations.

## Methods

### Ethics Statement

This study complies with the Declaration of Helsinki and the Walsall Local Research Ethics Committee reviewed and approved the protocol (05/Q2708/45). Verbal and written consent was obtained from all participants.

#### Study population

The design and protocol of the E-ECHOES study has previously been published [19]. In brief, this was a cross-sectional population survey of a sample of SA (i.e. those originating from India, Pakistan or Bangladesh) and AC (i.e. those originating from the Caribbean and sub-Saharan Africa) residents of Birmingham aged 45 years and over. The majority of the SA and AC groups in the UK reside in metropolitan areas particularly inner cities such as Birmingham [17].

**Figure 1:**
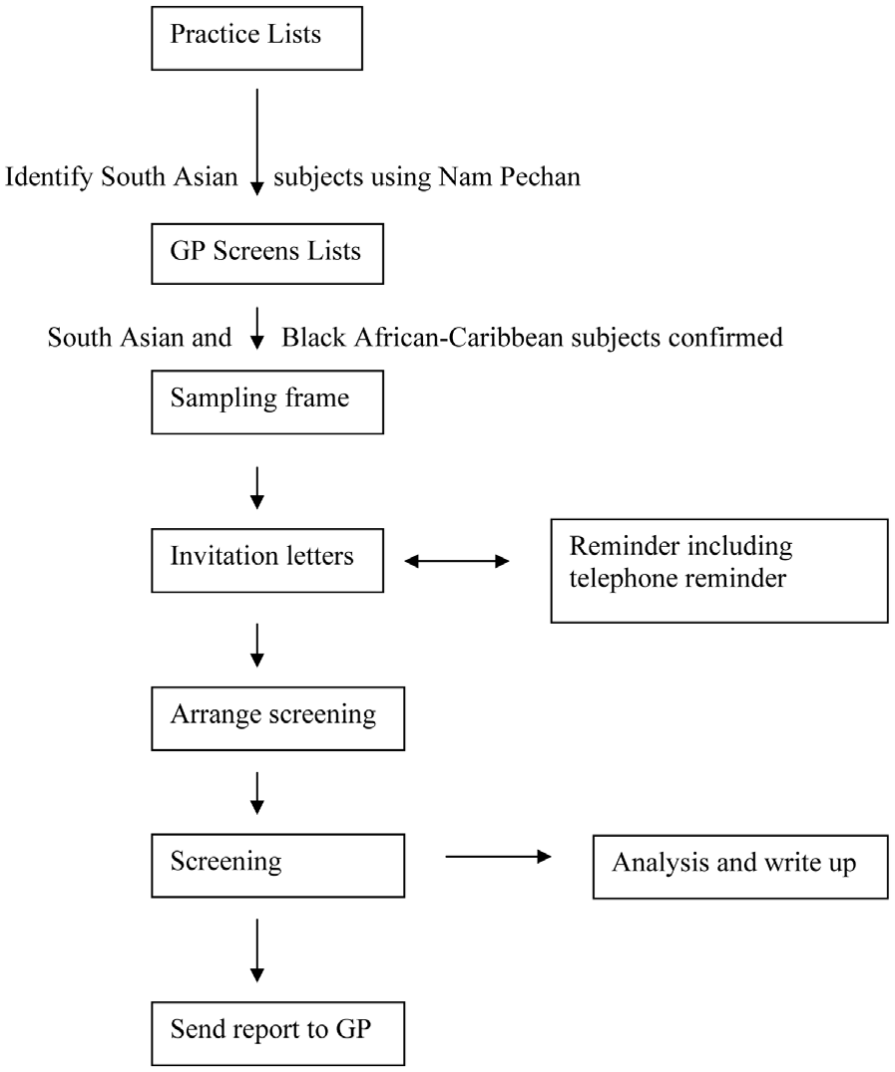
Flow of participants through study.

Recruitment was undertaken from September 2006 to August 2009 from 20 primary care centres. This entailed a two-staged process with an initial sample of primary care centres known to have high proportion of these minority ethnic patients and then a sample using the practice age-sex register. As ethnic group collection is not routinely collected in primary care, we used multiple methods to identify the subjects. Potential SAs were identified using the Nam Pechan software based upon subject name and visual inspection by PSG [20]; and for AC subjects practice staff were consulted (see Figure 1). The general practitioner then reviewed the lists to ensure that only SA and AC subjects were included and excluded any whom they considered it inappropriate to approach; for example, due to terminal illness or dementia.

Potential subjects were mailed an invitation letter, a reminder and telephoned up to 3 times inviting them to participate in the study. All potentially eligible subjects were asked their ethnic group before booking an appointment.

#### Measurements

To maximise recruitment, we invited all eligible subjects to attend for an assessment at their local primary care centre. Data obtained included an interview-administered comprehensive questionnaire, full physical examination, ECG and echocardiogram. Interpreters were used as required. Self-reported diagnoses were confirmed with practice medical notes.

A resting 12 lead ECG was recorded and reported by the clinical research team, and a random sample was reported independently by 3 consultant cardiologists (RD, MD, GL). Echocardiography was performed within the general practice surgeries using a portable VIVID i machine (GE Healthcare, Chalfont St Giles, UK), Chamber dimensions were obtained from the parasternal windows, and the presence and degree of left ventricular hypertrophy noted. Left ventricular function was measured objectively using an area-length method from the apical four-chamber view. In cases where an objective measurement of left ventricular ejection fraction (LVEF) was not possible, a qualitative assessment was made, that is, definite impairment (LVEF<40%), borderline (40–50%) and preserved (>50%), consistent with the investigators' normal clinical practice (RD, MD, GYHL). Valve disease was assessed semi-quantitatively and recorded, along with any other abnormalities. Parameters of diastolic function (mitral valve E∶A ratio; E wave deceleration time; and isovolumic relaxation time, and Pulsed Wave Tissue Doppler studies of movement of the mitral valve annulus, with derivation of the E∶e prime ratio) were also measured. Heart Failure, both systolic and with preserved ejection fraction (HFPEF), was defined using explicit criteria [13].

### Quality control measures

The research team were given training at the start of the project on administering the questionnaire; performing physical examination, followed by an ECG and echocardiogram. The team followed written protocols that included a number of quality control checks, including re-reporting by senior cardiologists (RD, MD) of all abnormal echocardiograms and a sample of those reported by the research fellow as normal. Both RD and MD undertook quality assurance in the ECHOES study [7].

### Sample Size and statistical analysis

Full details of the sample size and analysis plan have previously been provided in the E-ECHOES protocol paper [19]. Briefly, we aimed to recruit 3000 SA and 2000 AC patients. The precision of estimation of prevalence is dependent upon the number of subjects and the prevalence rate. The principal aims of this study were to estimate the prevalence of LVSD and heart failure, and atrial fibrillation, in the SA and AC populations.

Descriptive analyses were performed on all study variables, describing rates as percentages, and continuous variables as medians and lower and upper quartiles. Prevalence estimates are described with 95% confidence intervals. For the comparisons of heart failure rates by survey, a test for interaction was used and confidence intervals presented [21]. Descriptive data were compared to those from the ECHOES study where LVSD was assessed amongst a predominantly White population [7]. Due to the small number of cases, age-sex adjustment was not conducted.

## Results

### Subjects

**Figure 2:**
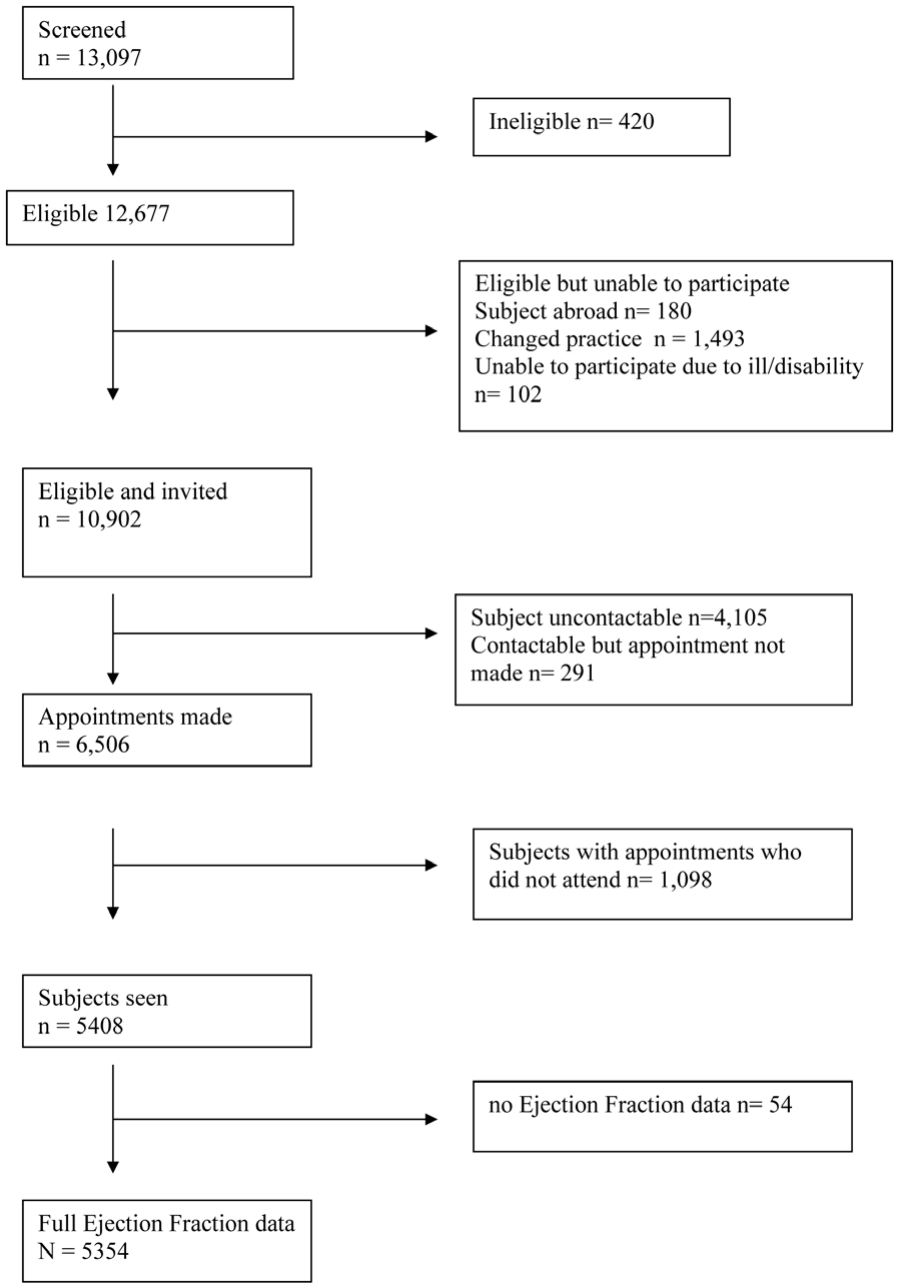
Study profile.

Of 13,097 subjects screened , 10,902 were eligible and invited and 420 (3.2%) did not meet the study inclusion criteria. 6,506 (59.7% of those eligible and invited had an appointment booked of which5,408 (49.6%) completing the screening process and 5,354 (%) having complete LVEF data (Figure 2).

Table 1 shows that for AC group, non-responders were likely to be younger males from less deprived areas in contrast SA non-responders who were likely to be males from deprived areas. The conclusions are the same for those subjects *potentially* eligible (Table S1) for this study (i.e. those unable to participate due to being abroad; change of practice or ill health/disability).

**Table 1:** Characteristics of responders and non-responders.

	Responders withEjection Fraction data	Non responders	Difference in Means (95% CI)	P Value
**South Asians (n,%)**	**3442(53.4)**	**3007(46.6)**		
Age at initial contact (mean [SD], years)	59.4 (10.4)	59.5 (11.2)	−0.0009 (−0.0054 to 0.0037)	0.71
Index of Multiple Deprivation 2007 (mean [SD])	49.4 (15.8)	52.6 (13.9)	−0.017 (−0.020 to −0.0129)	<.0001

			Odds Ratio (95% CI)	
Male (n, %)	1690 (49.1%)	1566 (52.1%)	1.12 (1.02 to 1.24)	0.02
**African Caribbean (n,%)**	**1912(42.9)**	**2541(57.1)**		
Age at initial contact (mean [SD], years)	62.5 (12.0)	59.7 (12.2)	0.0158 (0.0106 to 0.021)	<.0001
Index of Multiple Deprivation 2007 (mean [SD])	53.4 (12.6)	52.7 (14.1)	0.0076 (0.0026 to 0.0126)	0.0029

			Odds Ratio (95% CI)*	
Male (n, %)	854 (44.7%)	1302 (51.2%)	1.34 (1.18 to 1.52)	<.0001

Univariate analyses with practice as random effects;

*Odds ratio for response: female compared to male.

Index of Multiple Deprivation 2007 Score: higher scores indicate increased deprivation. [http://www.communities.gov.uk/communities/research/indicesdeprivation/].

Of those with complete LVEF data, 3442 (64.28%) were SA and 1912 (37.71%) were AC subjects. Subject demographics are summarised in Table 2. The median age of participants was 58.2 years (IQR, interquartile range: 51.0 to 70.0) of which 2810 (52.5%) were female. The average deprivation index was 58.2 for the whole cohort, with SA being lower (56.8) and AC being higher (64.0). The proportion of patients with hypertension or diabetes was relatively high in both groups (hypertension: 45.6% of SA and 57.9% of AC; diabetes 30.8% and 26.3%, respectively). Table S2 shows clinical characteristics of all subjects by ethnic group and ejection fraction.

**Table 2:** Clinical characteristics of all subjects by ethnic group.

	* SA Total (n = 3442)	AC Total (n = 1912)	p value
Age (mean [SD], years)	59.7 (10.4)	62.7 (12.0)	<0.0001
Male	1690 (49.10%)	854 (44.67%)	0.0019
Systolic blood pressure(Mean, SD, mm Hg)	139.51 (19.85)	144.32 (19.88)	<0.0001
Diastolic blood pressure(Mean, SD, mm Hg)	80.87 (10.91)	81.99 (10.79)	0.0003
Ever smoked	801 (23.27%)	826 (43.20%)	<0.0001
Consumes alcohol(occasionallyor regularly)	552 (16.04%)	1306 (68.31%)	<0.0001
BMI Median (IQR)	27.56 (24.89to 31.13)	28.99(25.89to 33.05)	
Index of Multiple Deprivation2007 Median, IQR	54.74(39.02to 61.39)	57.93 (46.25to 61.34)	<0.0001
New York HeartAssociation Class			<0.0001
I	154 (4.47%)†	82 (4.29%)†	0.7515
II	81 (2.35%)	42 (2.20%)	0.714
III	16 (0.46%)	8 (0.42%)	0.8075
IV	0 (0%)	0 (0%)	-
EQ-5D Median (IQR)	1 (0.848 to 1)	1 (1 to 1)	<0.0001
Hypertension	1570 (45.61%)	1106 (57.85%)	<0.0001
Angina	328 (9.53%)	87 (4.55%)	<0.0001
Myocardial infarction/ACS/Revascularisation(PCI/CABG)*	299 (8.69%)	64 (3.35%)	<0.0001
Heart Failure	46 (1.34%)	29 (1.52%)	0.5474
Diabetes	1060 (30.80%)	503 (26.31%)	0.0005
Peripheral artery disease	18 (0.52%)	26 (1.36%)	0.0015
Stroke/TIA	157 (4.56%)	67 (3.50%)	0.0554
ACE Inhibitors	818 (23.77%)	465(24.32%)	0.6486
Diuretics	639 (18.56%)	616 (32.22%)	<0.0001
Beta-blockers	470 (13.65%)	236 (12.4%)	0.1741
Calcium Antagonists	613 (17.81%)	716 (37.45%)	<0.0001
Aspirin	1054 (30.62%)	563 (29.45%)	0.3691
Warfarin	35 (1.02%)	34 (1.78%)	0.0239
Digoxin	13 (0.38%)	13 (0.68%)	0.1077
Lipid regulating drugs	1483 (43.09%)	731 (38.23%)	0.0006

SA South Asian; AC African-Caribbean; BMI Body Mass Index, TIA Transient Ischaemic Attack; EQ-5D EuroQol 5D instrument;

† New York Heart Association Class reported only in those with cardiac disease;

*includes 1 Singalese subject with no heart failure.

#### Prevalence of Left-Ventricular Systolic Dysfunction and Symptomatic Heart Failure

In the whole cohort, 59 subjects (1.1%; 95% CI 0.84 to 1.42%) had an LVEF<40% (Table 3). Of these 40 had symptoms of dyspnoea with NYHA≥2. Of the remaining 19 patients with an LVEFEF<40% four patients were treated with furosemide, so presumably had had previous symptoms of heart failure.

**Table 3:** Prevalence of Left Ventricular Systolic Dysfunction and Heart Failure (%).

	South Asian	Black African-Caribbean	All
	3442	1912	5354
**EF (%)**			
<40	42 (1.22%; 95% CI 0.88 to 1.65%)	17 (0.89%; 95% CI 0.52 to 1.42%)	59 (1.10%; 95% CI 0.84 to 1.42%)
40 to 50	41 (1.19%; 95% CI 0.86 to 1.61%)	16 (0.84%; 95% CI 0.48 to 1.36%)	57 (1.06%;95% CI 0.81 to 1.38%)
>50	3359 (97.59%; 95% CI 97.02 to 98.07%)	1879 (98.27%; 95% CI 97.58 to 98.81%)	5238 (97.83%; 95% CI 97.41 to 98.21%)
**LV Systolic Heart Failure**			
EF<40 and NYHA≥2	28 (0.81%; 95% CI 0.54 to 1.17%)	12 (0.63%; 95% CI 0.32 to 1.09%)	40 (0.75%; 95% CI 0.53 to 1.02%)

EF Ejection Fraction.

NYHA New York Heart Association.

In SA, 42 subjects (1.22%; 95% CI 0.88 to 1.65%) had an LVEF<40%, with mean age of 67.0 years(SD 9.9) of which 29 (69.05%) were male. In AC, 17 (0.89%; 95% CI 0.52 to 1.42%) had an LVEF<40% with a mean age of 72.4 years (SD 11.0), of which 15 (88.24%) were males. The prevalence of LVSD in the SA population is not statistically different from the prevalence observed in the AC population.

Table 4 shows that symptomatic heart failure occurred in 26 subjects with no underlying valve disease or atrial fibrillation.

**Table 4:** Heart failure in relation to associated valve disease and/or atrial fibrillation.

	EF<40, NYHA≥2		HFPEF, NYHA≥2		Total	
	SA	AC	SA	AC	SA	AC
Sinus rhythm with no valve disease	20 (0.58%)	6 (0.31%)	27 (0.78%)	10 (0.52%)	47 (1.37%)	16 (0.84%)
Atrial fibrillation without significant valve disease	3 (0.09%)	0 (0%)	0 (0%)	1 (0.05%)	3 (0.09%)	1 (0.05%)
Valve disease without AF	5 (0.15%)	4 (0.21%)	10 (0.29%)	4 (0.21%)	15 (0.44%)	8 (0.42%)
Atrial fibrillation and valve disease	0 (0%)	2 (0.10%)	1 (0.03%)	1 (0.05%)	1 (0.03%)	3 (0.16%)
Total	28 (0.81%)	12 (0.63%)	38 (1.10%)	16 (0.84%)	66 (1.92%)	28 (1.46%)

SA South Asian; AC African-Caribbean.

HFPEF heart failure with preserved ejection fraction.

NYHA New York Heart Association.

Numbers are proportions of total populations (SA, n = 3442; AC, n = 1912).

Of the 40 patients with an LVEF<40% and NYHA≥2 25 (62.5%) were prescribed an ACE inhibitor and 18 (45%) were prescribed a beta-blocker. Of the 19 patients with an LVEF<40% but without symptoms four patients were treated with furosemide (3 patients 40 mg daily; 1 patient 160 mg daily). Table 3 shows that the prevalence of systolic heart failure is not statistically different between the SA and AC groups.

### Point prevalence of atrial fibrillation

Overall, 51 subjects (0.95%; 0.71 to 1.25%) had atrial fibrillation: 26 (1.36%; 95% CI 0.89 to 1.99%) AC patients had evidence of atrial fibrillation with a mean CHADS2 score of 2.3 (SD 1.0whilst 25 (0.73%; 95% CI 0.47 to 1.07%) SA patients had evidence of atrial fibrillation with a mean CHADS2 score of 2.5 (SD 1.4) .

#### Left ventricular systolic dysfunction in high risk populations and a prior diagnosis of heart failure

Overall, 75 subjects (1.40%) reported a medical history of heart failure. Of the 59 patients identified with a LVEF<40%, 23 (39.0%) had a prior diagnosis of heart failure. A further 52 patients reported heart failure of which 13 patients had an EF of 40 to 50% and the remaining 39 patients had an LVEF>50%.

#### Heart failure with preserved ejection fraction (diastolic heart failure)

Table 4 shows causes of heart failure with 54 subjects (AC 16 ; SA 38) classified with definite heart failure with preserved ejection fraction. The majority (37) had no underlying valve disease or atrial fibrillation. All patients with definite heart failure with preserved ejection fraction had NYHA≥2.

## Discussion

In this first large community based epidemiological study, we have documented the prevalence of LVSD (SA: 1.22%; 95% CI 0.88 to 1.65%; AC: 0.89%; 95% CI 0.52 to 1.42%) and heart failure (SA: 0.81%; 95% CI 0.54 to 1.17%; AC: 0.63%; 95% CI 0.32 to 1.09%) amongst UK minority ethnic groups. In addition, we report the first epidemiological data on the prevalence of atrial fibrillation in these subjects (0.95%; 95% CI 0.71 to 1.25%).

The overall prevalence (0.75%) of HF was similar to that documented in primary care for the general population in England (0.7%) [22]. It was surprising to note that this was not higher given that SAs living in the UK, have a 50% greater risk of dying prematurely from coronary heart disease than the general population. A reanalysis of the data from our own study of acute HF admissions to a UK hospital [23] had suggested that the relative risk of HF in those aged 60–79 years was 3.1 (95% CI 1.9–4.9) for AC, and 5.2 (95% CI 3.7–7.4) for SA. The present community study does not support this, with a lower prevalence of HF in both ethnic groups. The lower rates of LVSD and HF in our cohort could conceivably reflect higher rates of myocardial infarction fatality though recent observational studies suggest otherwise [24], [25]. Of note, the misdiagnosis of HF is a possibility, given the high number of patients with normal ejection fraction that had diagnosis of heart failure, but levels are broadly consistent with those seen in the predominantly white community study [7]. As the latter study was undertaken more than 10 years ago (between 1995–1999), there needs to be caution in comparing these data. Another plausible explanation is the reduction of cardiovascular risk factors during the past decade that has prevented the occurrence of HF amongst these groups [26]. Further, nearly two-third of the subjects were taking drugs such as diuretics and ACE inhibitors and prescription items for ACE inhibitors and AIIRAs have been increasing that may potentially have alleviated progression to heart failure [27], [28].

We found that the mean age of HF amongst SA and AC groups is similar to that amongst the White population [7]. In contrast, African Americans have been reported to have a higher prevalence of HF than other groups and they present at a younger age [4]. The aetiology of HF is not known amongst the latter group and is unlikely to be due to coronary artery disease as in our study sample [29]. However, there is a higher prevalence of hypertension amongst the AC communities, which is a major cause of HFPEF or so-called ‘diastolic dysfunction’ [1]. This suggests that the HF epidemiology of US African Americans and UK AC populations may be different [30]. We also report for the first time prevalence estimates of HFPEF amongst these minority ethnic communities as assessed by robust Doppler criteria and observed that HFPEF appears higher amongst SA than the AC groups. Most management strategies have focussed on heart failure with LVSD and management of HFPEF remains elusive [1], [30].

The prevalence of hypertension and diabetes in participants with LVSD appears higher in these 2 minority ethic groups than in the White population (prevalence hypertension SA 78.6%; AC 76.5%; White British 39%; prevalence diabetes SA 40.5%; AC 58.8%; White 15%) [7]. The myocardial ischaemia rate, however, is comparable amongst the SA (54.8%) and White (53%) groups [7] and much lower amongst the AC group (41.2%). The latter is in line with data from the US where HF in Black Americans is less likely to be due to coronary heart disease than that in whites [1]. Galasko et al [31] showed that in a community sample that there was no difference in prevalence of LVSD between the white and non-white ethnic groups. However only 188 SA subjects were included with a higher cut-off LVEF<45%. It was also surprising to note the low numbers of HF associated with valve disease in our cohort given the burden of rheumatic heart disease in India [32] and our subjects migrating to the UK as young adults (median age 22 years for SA [IQR 17–30] and AC [IQR 16–28]).

Some data on HF in hospitalized ethnic groups are available from the UK. In a study conducted in Leicestershire involving 5789 consecutive patients [33], admission rates for HF were higher among SA patients than white patients. In this cohort, SA patients were younger and had more diabetes than white patients. In our analysis of acute admissions with HF to a district general hospital serving a multiracial population, we observed a higher *short term* (in-patient) mortality amongst the white European patients – with the mortality being 20.7% amongst Europeans, compared to 8.7% amongst AC, and 13.2% amongst SA – but the differences did not reach statistical significance [34]. At 8 years' follow-up, the total mortality was 90.5% amongst whites and 87.0% amongst non-whites (Log Rank test, p = 0.07) where the non-white patients had significantly better survival at all time points until 6 years, after which the survival curves started to converge [34]. In a matched historical cohort study of patients hospitalized for HF from Leicestershire [35], SA patients had similar rates of prior coronary artery disease but more often had hypertension and diabetes. As well as methodological issues these studies were conducted in secondary care, and have focused on particular ethnic groups by aggregating the groups, thus masking the differences that exist between minority ethnic groups, particularly the SA category [36]. Further community-based studies documenting the incidence of heart failure are needed.

### Atrial Fibrillation

The prevalence of AF was low in both ethnic groups, consistent with published small scale data. In the UK, one case-note review of known AF cases in 6 general practices, reported an AF prevalence of 0.6% [37], a figure consistent with our present observations from a large community screening study. Note that this does not include individuals with paroxysmal AF in sinus rhythm at the time of the investigation so may underestimate the true prevalence figure.

#### Strengths and Limitations of the study

Our study has several strengths: first it has a large sample of under-represented hard to reach groups [16] drawn from 20 centres increasing generalisability to these minority ethnic groups. Secondly, we used current guidelines on diagnosis of HF [13]. Thirdly, we used standard methods across both groups as in the previous study amongst the White population [7]. Fourthly, missing EF data was less than 1% in those having an echocardiogram.

There are potential limitations of our study. The response rate was low (49.6%), an increasingly common problem in epidemiological research, particularly amongst ethnic minority groups [38], [39], [40]. However this was comparable to similar heart failure community studies amongst minority [31] and white groups [7], [8], [41]. Indeed 6,506 (59.6%) make an appointment; 1,098 (16.9%) did not attend. (Fig. 2), 1,493 (26.7%) were confirmed to be no longer residing at the given address and the rest were not contactable despite the written and 3 telephone reminders. Further it is known that within inner city areas, due to the high mobility of the population it is difficult for primary care to maintain accurate registers [42].

Indeed these 2 minority ethnic groups are perceived to be reluctant to engage in research [16], [40], and we achieved the relatively high response by employing a number of strategies such as using multilingual research staff to telephone the subjects after the initial postal invitation; increasing awareness of the study within the community and engaging with practice staff. Further, comorbidities such as diabetes and hypertension were in line to those expected amongst these minority ethnic communities with, for example, diabetes being up to 5–6 times higher amongst SAs compared to the White group [17].

Also some subjects may not have attended for screening due to illness or disability and we have 102 subjects who stated this reason for non-attendance though none had a history of HF documented by the centre. We also minimised bias in analysis by ensuring data verification and locking the database before analysis. However, 1 Singhalese subject was included in the analysis and did not affect the main results (Table 2). iven the low prevalence of HF we were unable to evaluate predictors of HF as originally planned.

#### Conclusion

We have shown that the prevalence of HF and AF in the under-researched SA and AC minority communities in the UK appears comparable to that of the general population. HF and atrial fibrillation will continue to be a major cause of morbidity in all ethnic groups due to ageing of the population; and preventive strategies need to be identified and implemented.

## Supporting Information

Table S1 Responder characteristics compared to those potentially eligible.
(DOCX)

Table S2 Clinical characteristics of all subjects by ethnic group and ejection fraction.
(DOCX)
