# Supplementary material for: Prevalence of Heart Failure and Atrial Fibrillation in Minority Ethnic Subjects: The Ethnic-Echocardiographic Heart of England Screening Study (E-ECHOES)
Source: PLoS One. 2011 Nov 16;6(11):e26710. doi: 10.1371/journal.pone.0026710 (PMC3217919; doi:10.1371/journal.pone.0026710)
Supplement: Table S1 — Responder characteristics compared to those potentially eligible. (DOCX) [file pone.0026710.s001.docx]

**Table S1: Responder characteristics compared to those potentially eligible**

|  | **Responders with Ejection Fraction data** | **Non responders** | **Difference in Means (95% CI)** | **P Value** |
| --- | --- | --- | --- | --- |
| **South Asians (n,%)** | **3442(47.0)** | **3882(53.0)** |  |  |
| Age at initial contact (mean [SD], years) | 59.4 (10.4) | 59.7 (11.3) | -0.0042 (-0.0085 to 0.0002) | 0.0596 |
| Index of Multiple Deprivation 2007 (mean [SD]) | 49.41 (15.80) | 52.66 (14.09) | -0.0155 (-0.0189 to -0.0121) | <.0001 |
|  |  |  | **Odds Ratio (95% CI)** |  |
| Male (n, %) | 1690 (49.10%) | 1983 (51.08%) | 1.116 (1.016 to 1.226) | 0.0217 |
| **African Caribbean (n,%)** | **1912(35.7)** | **3441(64.3)** |  |  |
| Age at initial contact (mean [SD], years) | 62.5 (12.0) | 59.7 (12.3) | 0.0166 (0.0118 to 0.0213) | <.0001 |
| Index of Multiple Deprivation 2007 (mean [SD]) | 53.41 (12.63) | 52.85 (13.87) | 0.0073 (0.0023 to 0.0119) | 0.0020 |
|  |  |  | **Odds Ratio (95% CI)*** |  |
| Male (n, %) | 854 (44.67%) | 1758 (51.09%) | 1.37 (1.21 to 1.53) | <.0001 |

Univariate analyses with practice as random effects; * Odds ratio for response: female compared to male.

Index of Multiple Deprivation 2007 Score: higher scores indicate increased deprivation [http://www.communities.gov.uk/communities/research/indicesdeprivation/].

Note that non responders include all subjects potentially eligible for this study (including those unable to participate due to being abroad; change of practice or ill health).
