# Supplementary material for: Prevalence of Heart Failure and Atrial Fibrillation in Minority Ethnic Subjects: The Ethnic-Echocardiographic Heart of England Screening Study (E-ECHOES)
Source: PLoS One. 2011 Nov 16;6(11):e26710. doi: 10.1371/journal.pone.0026710 (PMC3217919; doi:10.1371/journal.pone.0026710)
Supplement: Table S2 — Clinical characteristics of all subjects by ethnic group and ejection fraction. (DOCX) [file pone.0026710.s002.docx]

**Table S2: Clinical characteristics of all subjects by ethnic group and ejection fraction**

| **Characteristic** | *** SA Total (n=3442)** | **SA Ejection Fraction <40% (n=42)** | **AC Total (n=1912)** | **AC Ejection Fraction <40% (n=17)** |
| --- | --- | --- | --- | --- |
| Age (mean [SD], years) | 59.7 (10.4) | 67.0 (9.9) | 62.7 (12.0) | 72.4 (11.0) |
| Male | 1690 (49.10%) | 29 (69.05%) | 854 (44.67%) | 15 (88.24%) |
| Systolic blood pressure (Mean, SD, mm Hg) | 139.51 (19.85) | 137.89 (21.28) | 144.32 (19.88) | 141.29 (25.81) |
| Diastolic blood pressure (Mean, SD, mm Hg) | 80.87 (10.91) | 78.12 (13.04) | 81.99 (10.79) | 78.26 (15.88) |
| Ever smoked | 801 (23.27%) | 13 (30.95%) | 826 (43.20%) | 7 (41.1 8%) |
| Consumes alcohol (occasionally or regularly) | 552 (16.04%) | 7 (16.67%) | 1306 (68.31%) | 9 (52.94%) |
| BMI Median (IQR) | 27.56  (24.89 to 31.13) | 26.12 (24.39 to 30.35) | 28.99  (25.89 to 33.05) | 26.17 (23.41 to 34.51) |
| Index of Multiple Deprivation 2007 Median, IQR | 54.74  (39.02 to 61.39) | 46.125 (33.01 to 63.87) | 57.93  (46.25 to 61.34) | 58.22 (54.26 to 61.34) |
| New York Heart Association Class |  |  |  |  |
| I | 154 (4.47 %) † | 7 (16.67%) † | 82 (4.29%) † | 3 (17.65%) † |
| II | 81 (2.35 %) | 18 (42.86%) | 42 (2.20%) | 7 (41.18%) |
| III | 16 (0.46%) | 10 (23.81%) | 8 (0.42%) | 5 (29.41%) |
| IV | 0 (0%) | 0 (0%) | 0 (0%) | 0 (0%) |
| EQ-5D Median (IQR) | 1 (0.848 to 1) | 0.823 (0.639 to 1) | 1 (1 to 1) | 0.848 (0.587 to 1) |

| Hypertension | 1570 (45.61%) | 33 (78.57%) | 1106 (57.85%) | 13 (76.47%) |
| --- | --- | --- | --- | --- |
| Angina | 328 (9.53%) | 19 (45.24%) | 87 (4.55%) | 4 (23.53%) |
| Myocardial infarction/ACS/  Revascularisation(PCI/CABG)* | 299 (8.69%) | 23 (54.76%) | 64 (3.35%) | 7 (41.18%) |
| Heart Failure | 46 (1.34%) | 14 (33.33%) | 29 (1.52%) | 9 (52.94%) |
| Diabetes | 1060 (30.80%) | 17 (40.48%) | 503 (26.31%) | 10 (58.82%) |
| Peripheral artery disease | 18 (0.52%) | 1 (2.38%) | 26 (1.36%) | 1 (5.88%) |
| Stroke/TIA | 157 (4.56%) | 5 (11.90%) | 67 (3.50%) | 2 (11.76%) |
| ACE Inhibitors | 818 (23.77%) | 24 (57.14%) | 465(24.32%) | 10 (58.82%) |
| Diuretics | 639 (18.56%) | 18 (42.86%) | 616 (32.22%) | 11 (64.71%) |
| Beta-blockers | 470 (13.65%) | 17 (40.48%) | 236 (12.4%) | 6 (35.29%) |
| Calcium Antagonists | 613 (17.81%) | 9 (21.43%) | 716 (37.45%) | 5 (29.41%) |
| Aspirin | 1054 (30.62%) | 26 (61.90%) | 563 (29.45%) | 10 (58.82%) |
| Warfarin | 35 (1.02%) | 6 (14.29%) | 34 (1.78%) | 1 (5.88%) |
| Digoxin | 13 (0.38%) | 4 (9.52%) | 13 (0.68%) | 1 (5.88%) |
| Lipid regulating drugs | 1483 (43.09%) | 33 (78.57%) | 731 (38.23%) | 11 (64.71%) |

SA South Asian; AC African-Caribbean; BMI Body Mass Index, TIA Transient Ischaemic Attack; EQ-5D EuroQol 5D instrument † New York Heart Association Class reported only in those with cardiac disease

* includes 1 Singalese subject with no heart failure
